# Immunofluorescence and High-Resolution Microscopy Reveal New Insights in Human Globozoospermia

**DOI:** 10.3390/ijms23031729

**Published:** 2022-02-02

**Authors:** Paula Sáez-Espinosa, Laura Robles-Gómez, Leonor Ortega-López, Jon Aizpurua, María José Gómez-Torres

**Affiliations:** 1 Departamento de Biotecnología, Facultad de Ciencias, Universidad de Alicante, 03690001 Alicante, Spain; paula.saez@ua.es (P.S.-E.); laura.robles@ua.es (L.R.-G.); 2 IVF Spain, Reproductive Medicine, 03540001 Alicante, Spain; l.ortega@ivf-life-group.com (L.O.-L.); j.aizpurua@ivf-spain.com (J.A.); 3 Cátedra Human Fertility, Facultad de Ciencias, Universidad de Alicante, 03690001 Alicante, Spain

**Keywords:** immunofluorescence, morphology, round-headed spermatozoa, sperm parameters, transmission electron microscopy

## Abstract

Globozoospermia is a rare and severe type of teratozoospermia characterized by the presence of round-headed, acrosomeless spermatozoa with cytoskeleton defects. Current data support a negative relationship between globozoospermia and intracytoplasmic sperm injection (ICSI) outcomes, revealing the need to perform exhaustive studies on this type of sperm disorder. The aim of this study was to evaluate different structural, functional and molecular sperm biomarkers in total globozoospermia with proper embryo development after ICSI. The combination of field-emission scanning electron microscopy (FE-SEM) and transmission electron microscopy (TEM) allowed us to identify and correlate eight morphological patterns with both types of microscopy. Additionally, results reported a high percentage of coiled forms, with cytoplasmic retentions around the head and midpiece. By fluorescent microscopy, we detected that most of the sperm showed tubulin in the terminal piece of the flagellum and less than 1% displayed tyrosine phosphorylation in the flagellum. Moreover, we did not detect chaperone Heat shock-related 70 kDa protein 2 (HSPA2) in 85% of the cells. Overall, these findings provide new insights into globozoospermia, which could have potential implications in improving sperm selection methods for assisted reproductive techniques.

## 1. Introduction

Globozoospermia is a severe form of teratozoospermia characterized by the presence of round-headed spermatozoa [1,2]. Two types of globozoospermia have been described: total globozoospermia (type I), when 100% of the sperm have a round head and no acrosomes; and partial globozoospermia (type II), when less than 100% of the sperm are affected [2]. The failure of spermatid differentiation to spermatozoa during spermiogenesis causes morphological aberrations such as coiled flagellum around the nucleus in sperm patients [3], disorganized mid-pieces [4] or the separation of nuclear membranes [5]. Other maturation defects such as persisting residual cytoplasmic droplets surrounding the head or the midpiece have also been reported [3]. These sperm morphology abnormalities seem to be one of the most important indications of low sperm quality in globozoospermia [2]. However, the consensus of morphologic criteria in globozoospermia is difficult to achieve due to the great heterogeneity of forms observed. Thus, strict morphological studies are needed in round-headed spermatozoa to identify additional structural sperm defects.

Morphological alterations in globozoospermia are further related to molecular aspects [3]. For instance, previous studies have demonstrated an increased percentage of DNA abnormalities compared to fertile controls using the terminal-deoxynucleotidyl transferase-mediated nick end labeling (TUNEL) assay [6,7]. In addition, several studies about the relationship between globozoospermia and acrosome development resulted in either absent or hypoplastic acrosomes [8,9]. Some additional studies focusing on chromatin structure defects in globozoospermia are available in the literature separately [7,10,11]. Nevertheless, the vast majority of studies are case reports where only one individual is examined, and therefore results remain scarce, partial and scattered. In this context, the reduced fertilization and birth rates following intracytoplasmic sperm injection (ICSI) in this patient group [12,13] enforce global and interrelated studies.

The identification of novel sperm quality biomarkers would facilitate the diagnosis of globozoospermia and improve the selection of more effective treatments. Thereby, among the potential candidate biomarkers are parameters that have previously been related to fertilization capacity in non-globozoospermic patients. These include the distribution of structural molecules like α-tubulin [14] and molecules involved in sperm–egg recognition such as the chaperone Heat shock-related 70 kDa protein 2 (HSPA2) [15] or the identification of functional markers of sperm capacitation such as tyrosine phosphorylation [16].

Considering the above, we present a comprehensive screening of new structural, functional and molecular quality sperm biomarkers in total globozoospermia with adequate embryonic development using ICSI.

## 2. Results

**Figure 1:**
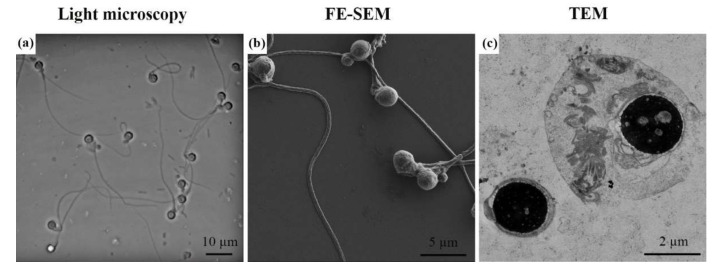
Globozoospermia micrographs from different types of microscopy: (**a**) light microscopy, (**b**) field-emission scanning electron microscopy (FE-SEM), and (**c**) transmission electron microscopy (TEM).

**Figure 2:**
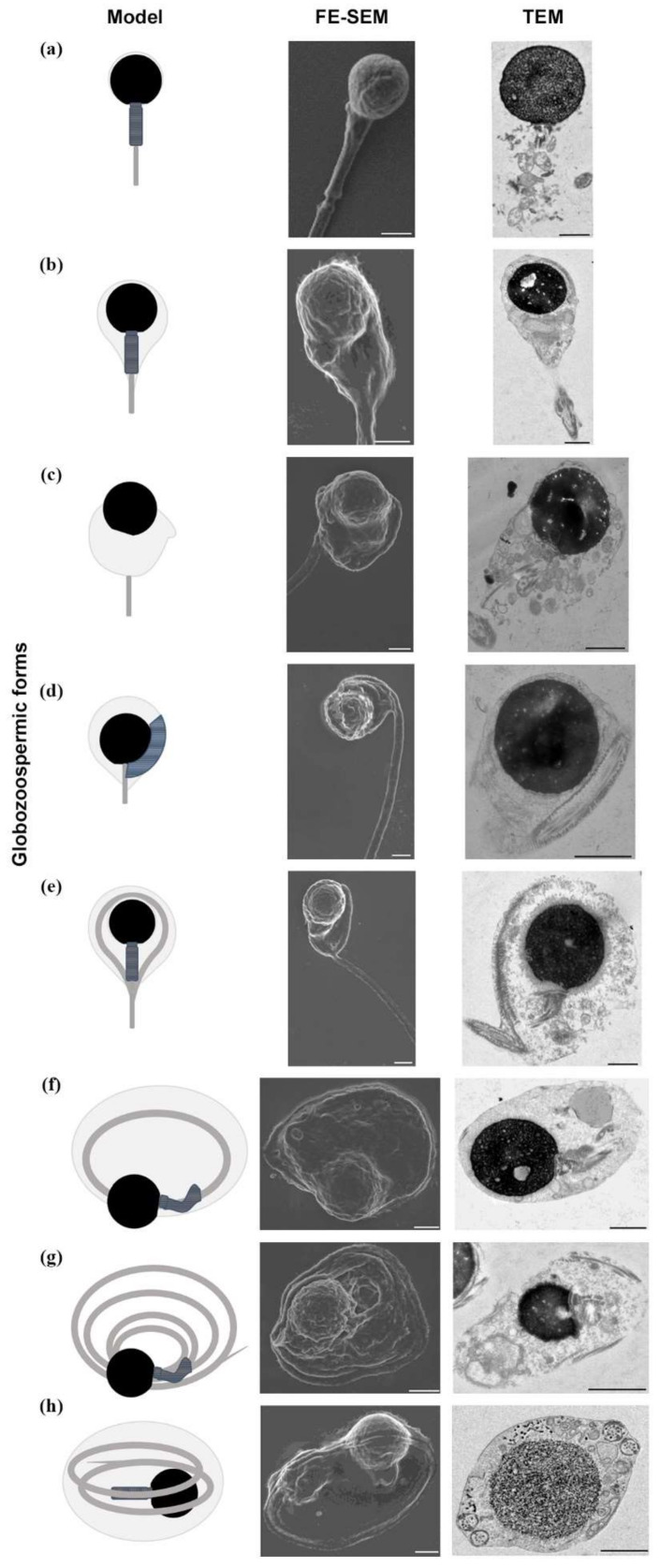
Morphological and ultrastructural micrographs of different round-headed sperm observed in this study using field-emission scanning electron microscopy (FE-SEM) and transmission electron microscopy (TEM), respectively. (**a**) Straight flagellum with no perinuclear cytoplasmic content (26.5%); (**b**) straight flagellum with cytoplasmic content around the midpiece and residual perinuclear cytoplasm (32%); (**c**) straight flagellum with large cytoplasmic droplet around the head and the midpiece (11.5%); (**d**) midpiece around the nucleus and flagellum starting to coil (7.5%); (**e**) flagellum around the head once and then going straight (3%); (**f**) flagellum totally around the nucleus inside a cytoplasmic droplet (10.5%); (**g**) total flagellum encirclements around the nucleus (5%); (**h**) total coiling many times around the nucleus (4%). Scale bar 1 µm common to all images.

Semen volume was 2 mL, sperm concentration in the ejaculate was 28 × 10^6^ spermatozoa/mL, total motility was 40% (progressive motility 30%) and sperm vitality evaluated using propidium iodide was 71%. The morphological analysis of the sperm by light microscopy did not detect normal forms. All spermatozoa had round heads, classifying the sample as total or type I globozoospermia [4]. We also observed several anomalies, such as coiled flagellum around the head or notable cytoplasmatic droplets. FE-SEM and TEM micrographs confirmed the presence of aberrances previously detected by light microscopy (Figure 1 and Figure 2).

Specifically, detailed observation by TEM showed that the round heads were linked with a round-shaped nucleus with frequent vacuolar areas (Figure 2b,f,g). Moreover, the round-shaped heads were devoid of acrosome and the chromatin was often characterized by granular texture, indicating genetic decondensation (Figure 2a,h). Accurate analysis both in FE-SEM and TEM allowed us to determine the percentages of the new different sperm anomalies detected (Figure 2). We assessed a total of eight distinctive morphological alterations that were described as follows: (a) straight flagellum with no perinuclear cytoplasmatic content and absence of acrosome (26.5%); (b) straight flagellum with mitochondria accumulated in the conical neck and presence of residual perinuclear cytoplasm (32%); (c) straight flagellum with large cytoplasmic droplet containing disorganized mitochondria around the head and the midpiece (11.5%); (d) midpiece around the nucleus and the flagellum starting to coil, possibly due to the dislocation of the connecting piece of the flagellum and the implantation fossa of the nucleus (7.5%); (e) flagellum around the head once and then going straight (3%); (f) inaccurate implantation of the nuclear fossa and the connecting piece of the flagellum facilitating total coiling inside a cytoplasmic droplet with disorganized organelles (10.5%); (g) total flagellum encirclements around the nucleus not included in a drop and midpiece with disorganized mitochondria (5%); (h) total coiling many times above the nucleus and midpiece with disorganized mitochondria (4%).

**Figure 3:**
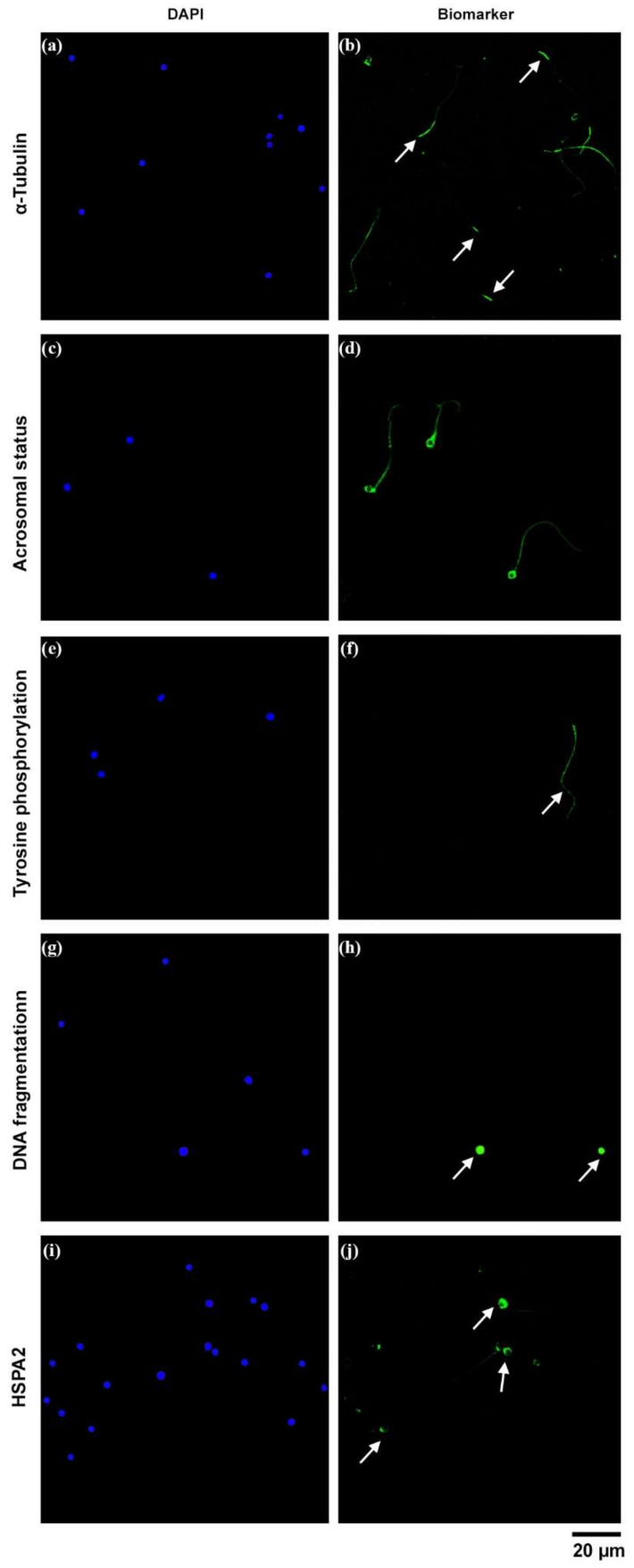
Micrographs of different human globozoospermia biomarkers taken by confocal microscopy. DAPI was used to detect the nucleus. (**a**,**b**) Distribution of α-tubulin. Note that most of the cells only showed α-tubulin label in the terminal piece (arrows). (**c**,**d**) Acrosomal status by *Pisum sativum agglutinin* label. (**e**,**f**) Tyrosine phosphorylation distribution. Positive tyrosine phosphorylation in sperm flagellum is indicated by an arrow. (**g**,**h**) DNA fragmentation. Positive TUNEL labeling implies DNA fragmentation (arrows). (**i**,**j**) Distribution of HSPA2. Note that only a few percentages of cells showed positive HSPA2 labeling around the sperm head (arrows).

Regarding tubulin staining, we observed two different flagellar structure patterns. Specifically, 33% of spermatozoa showed continuous labelling of α-tubulin in the flagellum, and in the remaining 67% the fluorescence appeared in the terminal piece of the flagellum (Figure 3a,b).

The assessment of acrosome reaction revealed that none of the spermatozoa presented fluorescence in the acrosomal region (Figure 3c,d). Moreover, we observed 0.5% of positive tyrosine phosphorylation in round-headed sperm, whereas 95.5% of the sperm analyzed did not exhibit positive fluorescence (Figure 3e,f).

Regarding DNA damage, we found that 11% of the sperm studied showed DNA fragmentation after the assessment of nucleus status (Figure 3g,h). Moreover, the study of the ultrastructure of the nucleus by TEM revealed low-electron-density regions due to decondensation of the genetic material (Figure 2). Finally, the immunolabeling of HSPA2 indicated that 15% of spermatozoa presented positive labeling around the head, whereas 85% did not show immunostaining (Figure 3i,j).

## 3. Discussion

Globozoospermia is only observed in <0.1% of infertile patients [13] which makes it challenging to study [17]. Nevertheless, low success rates using ICSI have led to a need for deeper knowledge of the novel characteristics of these several types of teratozoospermia. In this report, we performed a comprehensive description of certain structural, functional and molecular insights in one case of total globozoospermia treated with ICSI, resulting in a proper quality embryo but an unsuccessful pregnancy.

Some previous studies have described different conventional semen parameters in globozoospermic patients. However, the prevalence of isolated case reports in this group and the variety of techniques for evaluating seminal parameters hinder a consensus [2]. Our results showed that semen volume, sperm concentration, motility and viability were normally tending towards the lower reference limits of the WHO semen parameters [18]. Accordingly, previous case reports have described normal semen volume [10,19], sperm concentration [10,20] and total motility [10,19]. Nevertheless, some case studies have reported significantly reduced sperm concentration and motility in globozoospermic compared to normozoospermic patients [11,21,22,23,24]. The higher number of samples included in the case studies would explain the differences between sperm parameter results.

Morphology deserves special attention as a seminal parameter in globozoospermia. Thereby, we performed a detailed morphological description using light microscopy, TEM and FE-SEM. Papanicolaou staining allowed us to classify the sample as complete or type I globozoospermia [4]. Consequently, the percentage of normal morphology was 0%. This is following other studies using the same methodology [11,21,25] and Diff-Quik staining [10,19]. However, optical microscopy presented limitations to identifying morphological characteristics such as flagellum coiling levels. Conversely, FE-SEM allowed us to observe some specific abnormalities in a high percentage of spermatozoa from a globozoospermic patient, such as coiled flagella around the nucleus with notable cytoplasmic droplets. These findings are consistent with previous studies [3,26].

Focusing on flagellum morphology, we established an exhaustive and progressive identification of morphological forms, from incipient coiling of the midpiece around the head to full flagellum coiling. The coiled forms observed in this study are close to those described by Ricci et al., 2015 [26], indicating defective sperm maturation, as also shown in mice [27]. However, the rate of coiled spermatozoa was lower in our study in comparison with the previous research [26]. For instance, we observed more than 50% of total-straight flagellum forms, whereas the percentage of these forms according to other authors did not reach 10% [26]. Further, we identified additional forms in our study compared to Ricci et al., 2015 [26]. In this context, TEM allowed us to detail the flagellum coiling, especially whether it was included in a plasma membrane. Moreover, in accordance with previous reports, the coiled forms tended to show immaturity characteristics such as cytoplasmic retention [25]. Notable cytoplasmic droplets were present in our TEM images, providing the observation of several organelles inside.

Another important finding related to sperm flagellum structure was the characterization of two α-tubulin fluorescent patterns. To our knowledge, α-tubulin distribution in sperm from globozoospermic patients has not been described. Here, we observed in a minority of spermatozoa a pattern that consisted of continuous labelling throughout the flagellum. This pattern has been previously described in normozoospermia [28,29] and relates to proper sperm functionality [14]. However, in most spermatozoa we found a pattern characterized by a positive signal in the terminal piece of the flagellum in our study. This fluorescence distribution has been linked to flagellar structural alterations [30].

The relationship between morphologically abnormal sperm and nuclear alterations has been described. For instance, increased rates of decondensed chromatin were observed in some men with globozoospermia [7,31]. In our study, TEM allowed us to observe a granular texture in the nucleus of some spermatozoa, which is characteristic of uncondensed chromatin [25] and could be related to an abnormal chromatin structure in globozoospermia. In fact, abnormal chromatin packaging was demonstrated by aniline blue (AB) and acridine orange (AO) assays [25] and high histone/protamine ratios have been described in globozoospermic patients [10,23,32,33].

Certain reports also found increased DNA fragmentation rates in globozoospermia [7,11,21,23,34]. In our case, we assessed normal DNA sperm fragmentation (11%) using TUNEL as a testing method. Comparable DNA fragmentation indexes were obtained in previous reports [6,35,36]. However, a significant increase in DNA fragmentation identified using TUNEL has been described in globozoospermia compared with controls [21,36,37,38] that could at least partly explain the discouraging fertilization rates and pregnancy outcome using ICSI [22,39].

In general, the immunolocalization of additional biomarkers in globozoospermia is rather scarce. To the best of our knowledge, this is the first time that HSPA2 has been localized in sperm with globozoospermia. Due to the importance of the chaperone during egg–sperm recognition, the HSPA2 location has been recently described during in vitro capacitation [40]. In our study, we identified positive labeling for this protein around the head in only 15% of sperm from a globozoospermic patient. A similar pattern was previously observed in morphologically normal samples [15]. However, in most of the cells studied, we did not detect fluorescence. In this way, reduced expression of HSPA2 has been associated with abnormal morphology [41], cytoplasmic retention [41,42] or reduced fertility potential and pregnancy failure after in vitro fertilization (IVF) treatments [43]. The relationship between the low expression of HSPA2 and morphological defects could explain pregnancy failure in globozoospermia. In this regard, HSPA2 expression has proven to be significantly higher in fertile compared to infertile individuals [15].

In our study, only 0.5% of sperm showed positive tyrosine phosphorylation in the flagellum. This rate differs from the percentages of phosphorylation (~9%) observed in normozoospermic samples. On the other hand, a relationship between the α-tubulin distribution in the terminal piece and the absence of tyrosine phosphorylation in normozoospermic samples has been found [29]. Hence, the high percentage of round-headed spermatozoa that showed terminal piece tubulin distribution and the low rate of tyrosine phosphorylation observed in this study would suggest a possible flagellar structural or functional disorder.

Globozoospermia is further related to the deletion of the *DPY19L2* gene, contributing to the inaccurate stabilization of the acrosome [44]. However, a low percentage of spermatozoa that seemed to present a small bud of acrosome has been described and related with successful pregnancy [20]. In our TEM images, we confirmed the absence of acrosome or acrosomal buds. In accordance, the absence of fluorescence in the acrosomal region using PSA lectin was indicative that acrosome was absent. Shang et al. (2019) also used PSA-FITC as a marker for acrosome differentiation in spermatozoa from a globozoospermic patient, reporting the absence of acrosome [45].

The overall fertilization rate in globozoospermic men remains lower than 50% [17,46], suggesting the need to improve existing treatment techniques [47]. In our case, the fertilization rate using ICSI with OAC was 20%. In contrast, fertilization rates higher than 75% were achieved by complementing an ICSI technique with OAC [48,49] or oocyte electrical activation (OEA) [50]. Despite the low fertilization rate, we obtained an embryo whose development was adequate, arriving at a hatching blastocyst on day five. Moreover, we discarded the presence of aneuploidies by NGS, allowing the classification of the only embryo resulting from ICSI as euploid. In this regard, we demonstrated that although globozoospermic men had a statistically significantly higher rate of sperm fragmentation than fertile men, only a slight increase in aneuploidy rate compared with controls [34] was present. Unfortunately, no pregnancy was accomplished after embryo transfer. It is known that round-headed spermatozoa are capable of undergoing chromatin decondensation and form pronuclei after ICSI [51], but on the contrary, the aforementioned defects in chromatin integrity have been proposed as cause of later embryo implantation failure or spontaneous abortions [52,53]. Other studies have achieved successful pregnancy in a couple with partial globozoospermia without previous oocyte activation [54] and successful fertilization and pregnancy using supplemental techniques such as intra-cytoplasmic morphologically selected sperm injection (IMSI) [20] without previous artificial oocyte activation in total globozoospermia.

## 4. Materials and Methods

### 4.1. Experimental Design

**Figure 4:**
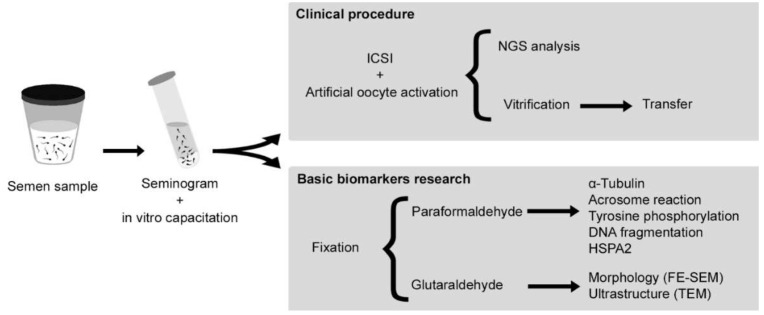
Experimental design followed in this research. ICSI, intracytoplasmic sperm injection; NGS, next-generation sequencing; HSPA2, chaperone Heat shock-related protein 2; FE-SEM, field-emission scanning electron microscopy; TEM, transmission electron microscopy.

After a basic seminogram and sperm in vitro capacitation, the semen sample was divided into two aliquots. One of them was designated for a clinical assisted reproductive procedure at IVF Spain, Reproductive Medicine, Alicante. The other one was designated for fixation and sperm biomarker analysis at the Departamento de Biotecnología (Universidad de Alicante, Spain). Specifically, a set of biomarkers were assessed: morphology by field-emission scanning electron microscopy (FE-SEM); ultrastructure using transmission electron microscopy (TEM); and α-tubulin, acrosome status, tyrosine phosphorylation, DNA fragmentation and HSPA2 by confocal microscopy (Figure 4). The entire study was done using a single seminal sample from a patient with globozoospermia. This research was approved by the Ethics Committee of the Universidad de Alicante (UA-2017-05-12) according to the principles of the Declaration of Helsinki.

### 4.2. Patient and Assisted Reproductive Technologies (ART)

A 38-year-old man and his 34-year-old partner requested ART (IVF Spain, Reproductive Medicine, Alicante, Spain) after a failed treatment with no fertilized oocytes in another fertility clinic. A semen sample was obtained by masturbation from the patient with his informed written consent. Standard semen analysis was performed according to the World Health Organization (WHO) criteria [18]. Sperm concentration and motility were determined with a Makler counting chamber and vitality was assessed using propidium iodide. Moreover, the semen sample showed 100% globozoospermia evaluated on 200 sperm cells with MorphoSlide Sperm (Vitromed, Jena, Germany) by light microscopy at 100× oil magnification. Thus, the couple proceeded to ICSI. Successively, the sample was prepared by density gradient (45/90% (*v/v*), centrifugation 300× *g* for 12 min) and then washed using Multipurpose Handling Medium-Complete (MHM-C, centrifugation 600× *g* for 5 min, IrvineScientific, Santa Ana, CA, USA). Supernatant was removed and sample was capacitated by swim-up using MHM-C (IrvineScientific , Santa Ana, CA, USA). MII oocytes were injected with spermatozoa from the globozoospermic patient, complementing the ICSI technique with oocyte activation by calcium ionophore (OAC) [55]. However, only one oocyte was fertilized (20%).

**Figure 5:**
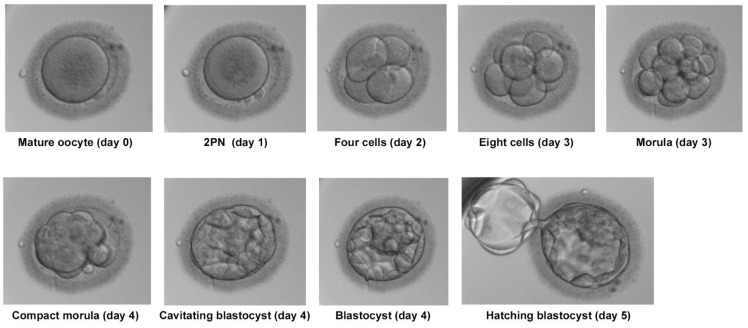
Development of the embryo resulting from ICSI with globozoospermic spermatozoa. Pictures were taken in a time-lapse incubator until day five.

The resulting embryo was cultured in continuous culture medium (CSC, IrvineScientific, Santa Ana, CA, USA) for five days, under hypoxic conditions at 5% (*v/v*) oxygen and 6% (*v/v*) CO_2_ at 37 °C in a time-lapse incubator (Geri, Merck, Darmstadt, Germany). The development of this embryo was adequate, the appearance of pronuclei occurred at the right time, divisions took place without fragmentation and qualities were 4–1, 8–1. The embryo achieved the morula stage and acquired Bt5AA status on day five (Gardner classification; Veeck, L) proceeding to perform embryo biopsy (Figure 5). The trophectoderm was sent to the genetics laboratory where it was analyzed using the Veri seq PGS Kit and the Mi seq (Illumina, San Diego, CA, USA). This method, based on the next-generation sequencing (NGS) technique, allows the analysis of the 46 chromosomes in a single assay. The result of this analysis indicated a euploid embryo. The embryo was vitrified with a medium intended for such use (IrvineScientific Vitrification, Santa Ana, CA, USA) for its subsequent transfer, but ultimately pregnancy was not achieved.

### 4.3. Fixation

After ICSI was performed, the excess of sperm sample was divided into two aliquots for fixation. Thus, one part of the sample was fixed in 2% (*v/v*) paraformaldehyde (Electron Microscopy Sciences, Hatfield, PA, USA) and the other in 2% (*v/v*) glutaraldehyde (Essentials for Microscopy Ltd. CO, UK) for 1 h at 4 °C. Then, paraformaldehyde and glutaraldehyde were replaced with phosphate-buffered saline without calcium or magnesium, pH 7.4 (PBS, Biowest, Nuaillé, France), and the samples were stored at 4 °C until use.

### 4.4. Morphological and Ultrastructural Characterization

A comprehensive study of morphological features was performed using FE-SEM. For FE-SEM analysis, a total of 5 μL of glutaraldehyde-fixed sperm suspension was placed on a round coverslip with a diameter of 12 mm and air-dried [56]. At that time, the coverslip was rehydrated three times with PBS for 5 min. Then, the coverslip was critical point dried and glued to the stubs with carbon adhesive tape for FE-SEM before being carbon sputtered (SCD 004 Sputter Coater; Bal-Tec AG, Balzers, Liechtenstein). Sperm morphology was examined using a Zeiss Merlin VP Compact FE-SEM (Zeiss, Oberkochen, Germany) with a selected voltage of 2 kV, EHT (extra-high tension) mode and Inlens Duo signal. Optimal conditions were found which gave the most stable, high-contrast surface images. Two hundred cells were evaluated for different sperm forms and their percentage, and digital micrographs were recorded at 10,000× magnification (1024 × 768 pixels) from the different forms determined.

For TEM analysis, an aliquot of the glutaraldehyde-fixed sample was embedded in blocks of 2% (*w/v*) agar (Sigma-Aldrich^®^, St. Louis, MO, USA) approximately 5 mm × 5 mm × 1 mm in size. A postfixation in 1% (*v/v*) osmium tetroxide (Electron Microscopy Sciences) in PBS was performed during 1 h at room temperature and rinsed in PBS. Subsequently, the sample was incubated in 0.5% (*v/v*) uranyl acetate for 1 h at room temperature and, after washing in PBS, it was dehydrated in an ascending series of ethanol concentrations immersed in propylene oxide (Electron Microscopy Sciences) for solvent substitution. Finally, the sample was embedded in epoxy resin EPON-812 (Electron Microscopy Sciences). Ultrathin sections were cut with a diamond knife and double-contrasted with uranyl acetate 5% (*v/v*) and lead citrate 2.5% (*v/v*). These were examined under a JEOL JEM-1400 Plus transmission electron microscope (Akishima, Tokyo, Japan) equipped with a Gatan Orius digital camera (Gatan, Pleasanton, CA, USA) for image capture at 15,000× magnification.

### 4.5. Functional and Molecular Aspects

The evaluation of the α-tubulin distribution was performed following previous protocols [29]. Briefly, a volume of 5 µL paraformaldehyde-fixed sperm suspension was placed on a coverslip. Once dry, the coverslip was washed three times in PBS and permeabilized by incubation in 0.1% (*v/v*) Triton X-100 for 10 min. Then, spermatozoa were incubated with 2% (*w/v*) BSA-PBS (bovine serum albumin, Sigma-Aldrich^®^, St. Louis, MO, USA) for 30 min, and then an anti-α-tubulin antibody (Sigma-Aldrich^®^) at 40 µg/mL for 1 h. The coverslip was washed with PBS thrice and a secondary anti-mouse IgG antibody conjugated to Alexa Fluor 488 (Jackson ImmunoResearch, Ely, UK) at 5 µg/mL for 1 h. Finally, the coverslip was washed three times with PBS and mounted with Vectashield^®^ and 4’,6-diamidino-2-phenylindole dihydrochloride (DAPI, Vector Laboratories, Burlingame, CA, USA).

For the assessment of spontaneous acrosomal reaction, a total of 5 µL of paraformaldehyde-fixed sperm suspension was placed on a coverslip and permeabilized with methanol for 30 min. After air drying, the coverslip was washed thrice in PBS and unspecific binding was blocked by 2% (*w/v*) BSA-PBS for 30 min. The sample was incubated in a dark humid chamber with *Pisum sativum agglutinin* lectin conjugated to fluorescein-5-isothiocyanate (PSA-FITC, Sigma-Aldrich^®^, St. Louis, MO, USA) at 50 µg/mL for 1 h; following that, three washes in PBS were performed. The coverslip was then mounted with Vectashield^®^ and DAPI (Vector Laboratories, Burlingame, CA, USA).) [57].

A total of 5 μL of sperm paraformaldehyde-fixed suspension was deposited on a coverslip. When dry, the coverslip was washed thrice in PBS and permeabilized by incubation in 0.1% (*v/v*) Triton X-100 for 10 min. To prevent unspecific binding, spermatozoa were blocked with 2% (*w/v*) BSA-PBS for 30 min. Tyrosine phosphorylation was detected using an anti-phosphotyrosine primary antibody produced in mice (PY20, Sigma-Aldrich^®^, St. Louis, MO, USA) at 2 µg/mL for 1 h and a secondary anti-mouse IgG antibody conjugated to Cyanine ™3 (Jackson ImmunoResearch, Ely, UK) at 5 µg/mL for 1 h in the dark. Finally, the coverslip was washed again three times with PBS and then mounted with Vectashield^®^ and DAPI [57].

The terminal-deoxynucleotidyl transferase-mediated nick end labeling (TUNEL) assay was carried out to recognize DNA fragmentation using the In-Situ Cell Death Detection Kit: Fluorescein according to the manufacturer’s guidelines (Roche Diagnostics GmbH, Mannheim, Germany). First, 5 μL of fixed sample was deposited on a coverslip and then washed three times in PBS and permeabilized with 0.2% (*v/v*) Triton X-100 (Sigma-Aldrich^®^, St. Louis, MO, USA) for 5 min. The TdT-labeled nucleotide combination was added and incubated at 37 °C for 1 h in a dark humid chamber. The coverslip was then washed thrice in PBS and mounted with Vectashield^®^ with DAPI.

To evaluate the HSPA2 distribution, we modified the previously described [15]. A volume of 5 μL of paraformaldehyde-fixed sample was placed on a coverslip and air-dried. The sample was rehydrated in PBS thrice and permeabilized with Triton X-100 at 0.2% (*v/v*) in PBS for 10 min at room temperature. After permeabilization, the coverslip was incubated overnight with anti-HSPA2 (Sigma-Aldrich^®^, St. Louis, MO, USA) at 1 µg/mL in PBS-BSA. Then, the coverslip was washed three times in PBS for 5 min and it was incubated with Anti-rabbit-FITC antibody (Vector Laboratories) at 15 µg/mL PBS-BSA for 1 h in a dark humid chamber. Then, the sample was washed three times in PBS. Once dry, the coverslip was mounted with Vectashield^®^ with DAPI.

Fluorescent biomarkers were evaluated by a Confocal Laser Scanning Zeiss LSM 800 Microscope (Zeiss, Oberkochen, Germany) and Zeiss Imaging Software at the technical services of the Universidad de Alicante. Z-stacks sections (1040 × 1040 pixels) of the entire sperm were obtained using an oil 63× objective and 408 nm and 561 nm lasers. Then, the sections were reconstructed using ZEN 2.5 lite software (Zeiss). Controls were performed omitting the first antibody in tubulin, PY20 and HSPA2 biomarkers. In terms of acrosomal reaction and DNA fragmentation, lectin and TdT-labeled nucleotide were omitted, respectively.

## 5. Conclusions

Here, we report novel structural, functional and molecular features in sperm from a total globozoospermic patient whose ICSI resulted in one high-quality embryo but an unsuccessful pregnancy. It should be noted that the coordinated use of FE-SEM and TEM allowed us to distinguish and correlate eight morphological patterns with both microscopies. Overall, our outcomes provide a new perspective on globozoospermia, which could have potential significance in improving sperm selection methods for assisted reproductive techniques. Future complementary morphological and molecular studies are necessary to elucidate the cause of low fertilization rates after ICSI in globozoospermic patients.

## Data Availability

For further information about the data presented in this study, contact the corresponding author.
